# Three-Dimensional Structure of Cytochrome c Nitrite Reductase As Determined by Cryo-Electron Microscopy

**Published:** 2018

**Authors:** T. N. Baymukhametov, Y. M. Chesnokov, E. B. Pichkur, K. M. Boyko, T. V. Tikhonova, A. G. Myasnikov, A. L. Vasiliev, A. V. Lipkin, V. O. Popov, M. V. Kovalchuk

**Affiliations:** National Research Center «Kurchatov Institute», Akademika Kurchatova Sqr., 1, Moscow, 123182 , Russia; Bach Institute of Biochemistry, Research Center of Biotechnology of the Russian Academy of Sciences, Leninsky Ave., 33, bldg. 2, Moscow, 119071, Russia; Petersburg Nuclear Physics Institute named by B.P. Konstantinov of NRC «Kurchatov Institute», 188300 , Leningradskaya Oblast, Gatchina, mkr. Orlova roshcha, 1, Russia; Centre for Integrative Biology, Department of Integrated Structural Biology, Institute of Genetics and of Molecular and Cellular Biology, 67404, 1 rue Laurent Fries, Illkirch, France; University of California San Francisco Mission Bay, Genentech Hall, San Francisco, CA, 94158- 2517 , USA; Shubnikov Institute of Crystallography of Federal Scientific Research Centre «Crystallography and Photonics» Russian Academy of Sciences, Leninsky Ave., 59, Moscow, 119333, Russia

**Keywords:** single particle analysis, high-resolution cryo-electron microscopy, structural biology, cytochrome c nitrite reductase

## Abstract

The structure of cytochrome c nitrite reductase from the bacterium Thioalkalivibrio nitratireducens was determined by cryo-electron microscopy (cryo-EM) at a 2.56 Å resolution. Possible structural heterogeneity of the enzyme was assessed. The backbone and side-chain orientations in the cryo-EM-based model are, in general, similar to those in the high-resolution X-ray diffraction structure of this enzyme.

## INTRODUCTION

With the recent advances in cryo-electron microscopy (cryo-EM) associated with improvements in spatial resolution and a decrease in the lower limit of the molecular weight accessible through this technique, the method has begun to rival X-ray crystallography [1, 2]. Owing to the unique opportunity of extracting structural information on heterogeneous objects in a nearly native state and a rather simple sample preparation [3], cryo-EM has become a powerful tool in modern structural biology [4, 5]. Cryo-EM is currently the only method capable of addressing such challenges as the structure determination of difficult-to-crystallize membrane proteins and searching for different states of molecular complexes, the method being complementary to classical X-ray crystallography [6].

In this work, the three-dimensional structure of cytochrome *c *nitrite reductase from the bacterium *Thioalkalivibrio nitratireducens *(TvNiR) [7] was studied for the first time by single-particle analysis [8] – a cryo- EM method for protein structure determination. This enzyme catalyzes the reduction of nitrite to ammonia without the release of reaction intermediates from the active site [7]. In solution and in the crystalline state, the enzyme exists as a stable hexamer that possess a bipyramidal shape with a characteristic height of ~150 Å and a base of ~120 Å [9]. The hexamer boasts D3 symmetry and a molecular weight of ~360 kDa, due to which TvNiR is a very convenient object for cryo-EM. The goal of this work was to determine the structure of TvNiR by cryo-EM and compare it with the high-resolution X-ray structure determined earlier [9-11].

## EXPERIMENTAL

**Protein isolation and purification**

Native TvNiR was isolated and purified in two steps, according to a procedure described earlier [7]. Anion-exchange chromatography was performed on a 35-mL column prepacked with DEAE Sepharose Fast Flow at 4°C using a BioLogic LP system (BioRad, USA). The column was pre-equilibrated with 25 mM potassium phosphate buffer, pH 7.0. After loading of the extract and washing of the column with the same buffer, the protein was eluted with a linear gradient of 0–1.0 M NaCl. Gel filtration chromatography was performed on an AKTA FPLC system (Amersham Biosciences, USA) equipped with a SuperdexTM200 10/300 column equilibrated with 50 mM potassium phosphate buffer, pH 7.0, supplemented with 0.15 M NaCl. For further structural studies, the protein was concentrated to 10 mg/mL.

**Fig. 1:**
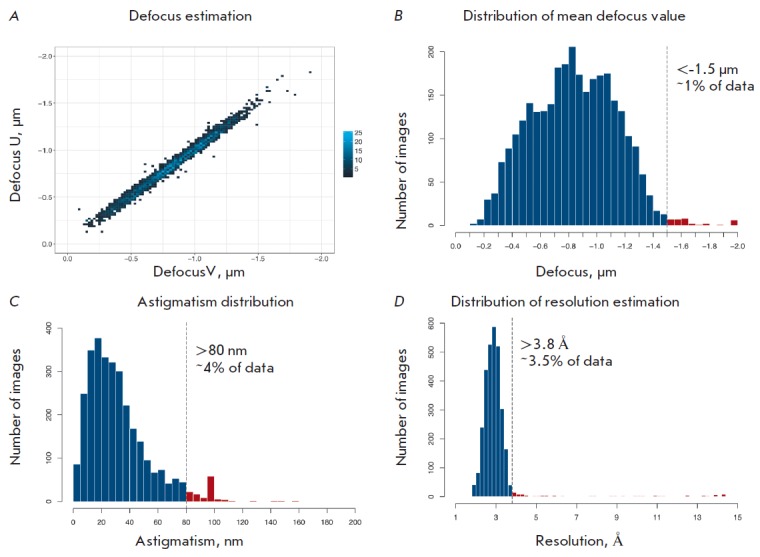
Principal characteristics of the initial data based on CTF parameters estimated with the Gctf program. A – the defocus distribution along two orthogonal axes; the color corresponds to the density of values at a specified coordinate. B–D – the distributions of the average defocus, astigmatism, and resolution assessment, respectively, along with the threshold values. The data with parameters in the ranges indicated in red were excluded from further processing

**Cryo-EM sample preparation**

In order to determine the optimal protein concentration in solution, concentrations in a range of 0.1 to 6.0 mg/mL were tested. The protein solutions were applied to Lacey Carbon 300 mesh copper grids (Ted Pella, USA). The experimental data were collected using Quantifoil R1.2/1.3 300 mesh grids (Quantifoil, Germany) coated with a carbon support film containing regular arrays of 1.2-μm circular holes spaced by 2.5 μm. The grids were glow-discharged for 30 s using a PELCO easyGlow system designed for hydrophilization (Ted Pella, USA) at a chamber pressure of 0.26 mbar and a current of 25 mA. A sample (3 μL, 6 mg/mL) was applied to the grids, and the grids were plunge-frozen in liquid ethane using a Vitrobot Mark IV vitrification device (Thermo Fisher Scientific, USA); the following parameters were set: blot force, 6; drain time, 1 s; temperature of the climate chamber, 4 °C; and humidity, 98 ± 2%.

**Table:** Summary of data collection parameters, cryo-EM map reconstruction, and structure refinement statistics for TvNiR

Data collection	
Accelerating voltage, kV	300
Magnification	75000x
Pixel size, Å	0.86
Exposure time, s	1.5
Number of image stacks	3055
Total dose per stack, e-/Å^2^	~100
Number of images per stack	30
Defocus range, µm	[-1.5; -0.5]
Defocus step size, µm	0.1
Cryo-EM map reconstruction	
Final number of particle images	33891
Symmetry group	D_3_
FSC_0.5_ (with masking / without masking)	2.86 / 3.19
FSC_0.143_ (with masking / without masking)	2.56 / 2.82
Resolution (average), Å	2.56
Structure refinement	
FSC_average_	0.8679
R_f_ (weighted, overall), %	28.70
Average B-factor, Å^2^	80.08
Rmsd bond lengths, Å^2^	0.018
Rmsd bond angles, deg	1.989
MolProbity score, %	2.55
All-atom clashscore, %	13.31
Good rotamers, %	91.68
Ramachandran outliers, %	0.19
EMDB code	EMD-0020

**Cryo-electron microscopy**

The grids were transferred at liquid nitrogen temperature to a Titan Krios cryo-electron microscope (Thermo Fisher Scientific, USA) equipped with a Schottky-type field emission electron gun (FEI XFEG, the Netherlands), a spherical aberration corrector (CEOS GmbH, Germany), and a CMOS-based Falcon II direct electron detector (Thermo Fisher Scientific, USA). A total of 3,055 image stacks were recorded in an automatic mode using the EPU package (version 1.9.1.16REL) (Thermo Fisher Scientific, USA) with a total exposure time of 1.5 s. The microscope was operated at an accelerating voltage of 300 kV and 75000×magnification corresponding to a pixel size of 0.86 Å at the specimen level, with objective lens defocused between –1.5 μm and –0.5 μm, with a step of 0.1 μm, using a total dose of ~100*e*-/Å2 evenly distributed across the image stack. The main data collection parameters are summarized in the *Table*. The principal data characteristics are shown in *Fig. 1A-D*.

**Fig. 2:**
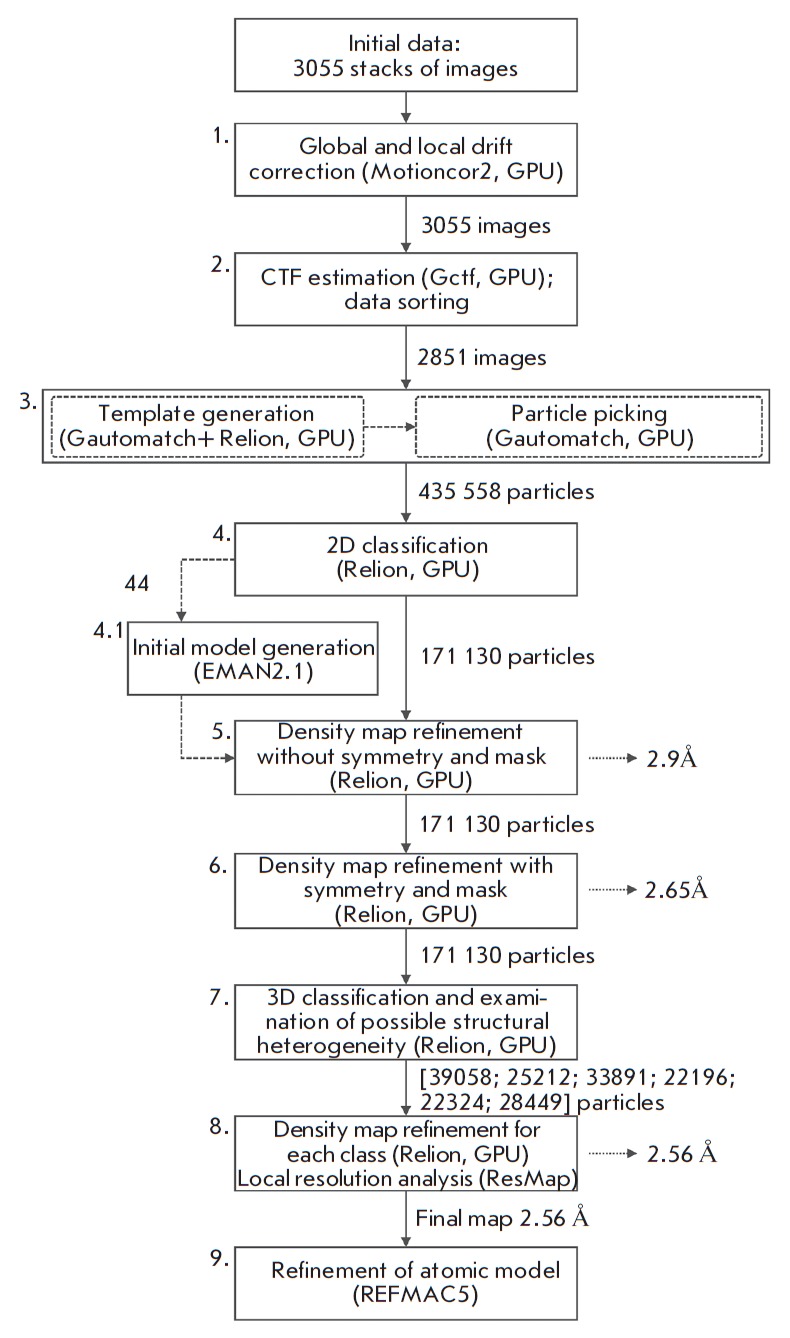
Main data processing steps

## RESULTS

**Cryo-EM map reconstruction**

The images were processed in several consecutive steps, presented in *Fig. 2*, using the computing resources of the Federal Collective Usage Center Complex for Simulation and Data Processing for Mega-Science Facilities at the National Research Centre Kurchatov Institute, equipped with Nvidia Tesla K80 graphics accelerators. The following image processing packages were employed: Motioncor2 (version 1.0.5) [12], Gctf (version 1.18) [13], Gautomatch (version 0.56) (K. Zhang, MRC Laboratory of Molecular Biology, Cambridge, UK, http://www.mrc-lmb.cam.ac.uk/kzhang/ Gautomatch), and Relion (version 2.1) [14]. All these packages were optimized for computations on graphics processors.

In the first step (*Fig. 2*), 3,055 initial image stacks were individually corrected for beam-induced motion using Motioncor2. The following two averaged and corrected image sets were obtained: (1) images filtered depending on the electron dose exposed to the sample (Dose Weighting) [15], which were used for classification and refinement processes; and (2) non-filtered images that were used to estimate contrast transfer function (CTF) parameters. In the second step (*Fig. 2*), the CTF parameters were estimated with the Gctf program. For each image, the information limit (resolution assessment), defocus, and astigmatism were estimated based on Thon ring fitting. The distribution’s tail points below the thresholds shown in *Fig. 1B-D* were excluded from further processing.

**Fig. 3:**
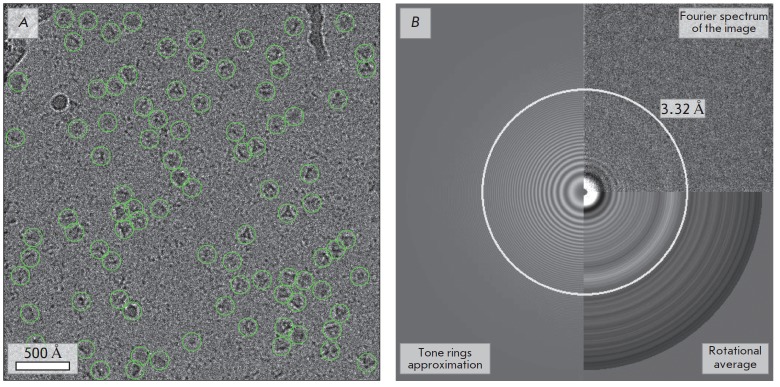
Preliminary data processing after correction for drift and estimation of the CTF parameters. A – an image with projections of the particles picked with Gautomatch; B – the Fourier spectrum of this image is shown in the upper-right section of the panel and the rotational average of the power spectrum is in the lower-right section of the panel; Thon ring fitting is presented in the left section. The circle, the radius of which in direct space corresponds to the assessment of the maximum resolution (3.32 Å), is highlighted

Therefore, 2,851 selected images with defocus, astigmatism, and resolution parameters not higher than –1.5 μm (in magnitude), 80 nm, and 3.8 Å, respectively, were used in all subsequent steps. A typical image after correction for drift is shown in *Fig. 3*.

In the third step (*Fig. 2*), particles were picked with Gautomatch. Initially, the procedure was applied for a subset of images recorded with a high degree of defocus. The 2D Gaussian function with a half-width corresponding to the characteristic size of an object was used as a template. The resulting set of particles was subjected to 2D classification in Relion. The classes containing projections of the object were utilized as templates to pick particles from the total data set. The resulting set contained 435,558 coordinates of possible particle positions.

**Fig. 4:**
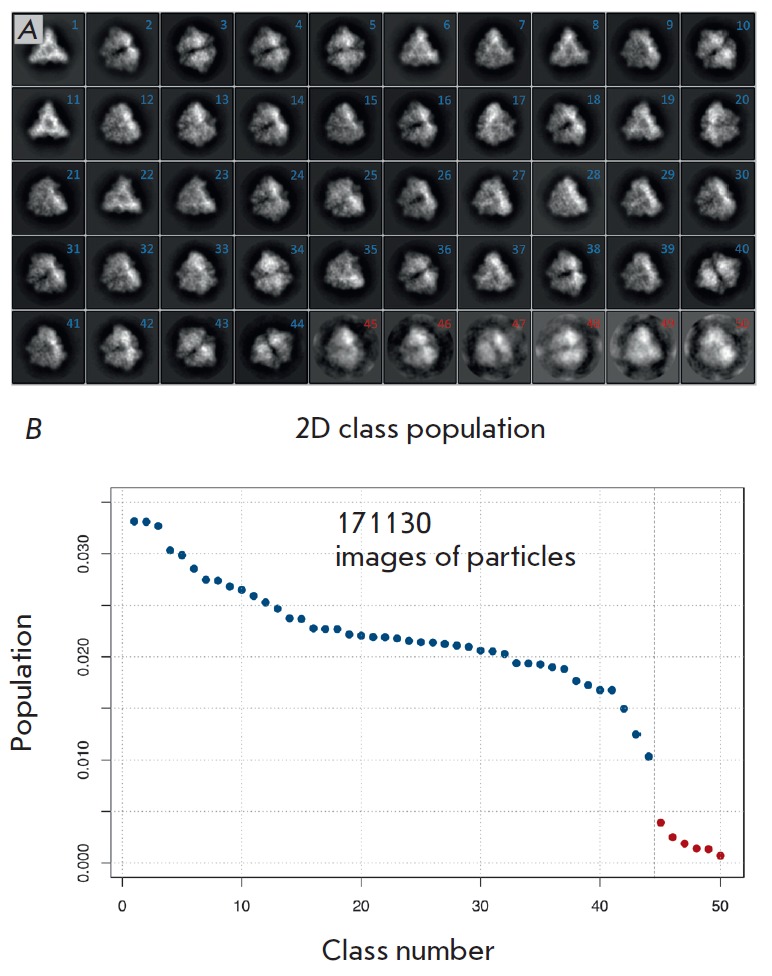
Fourth data processing step. A – images merged in each class after the second classification round. Particle images belonging to classes 1 to 44 (numbered in blue) were used for further analysis and density map construction. Particle images belonging to classes 45 to 50 (numbered in red), which led to overtraining of reconstruction algorithms, were excluded from further data processing; B – class population. The colors of the points correspond to the colors of the numbers in panel A

The fourth step (*Fig. 2*) involved two sequential rounds of classification. In the first round, the particle images were divided into 40 classes. Then, the images that were combined into classes not containing projections of the object or those depicting artifacts, such as ice crystals, surface contamination, and carbon edges, were excluded from the data set. In the second round, the remaining images were clustered into 50 classes, followed by the exclusion of the particles belonging to classes in which the boundaries of the object were not well-defined (*Fig. 4 A, B*).

After classification, 171,130 particle images containing structural information on the object were selected. A low-resolution initial model was built with the EMAN2 image processing package [16] by the Monte-Carlo method, taking into account the known symmetry of the object based on 44 projections obtained by averaging images present in the most populated classes, after the second classification round (*Fig. 4B*).

In the fifth step (*Fig. 2*), the low-resolution model was refined by reassigning the Euler angles and fitting projections of the object to the cryo-EM map in each sequential iteration of the EM algorithm implemented in Relion [17-19]. The refinement was carried out using the 3D auto-refine procedure without resorting to *a-priori *data on the symmetry of the object after postprocessing and applying the binary mask that defined the boundary conditions for the calculation of cross-correlation coefficients between two independently refined maps [20]. The resolution of the final map was 2.9 Å, as determined by the FSC = 0.143 criterion [21].

**Fig. 5:**
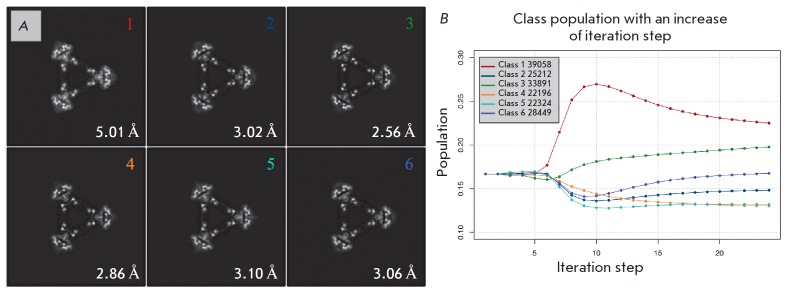
Results of 3D classification. A – central sections of the classes. Images from 1 to 44 (blue) were used for further analysis and density map reconstruction. Images from 45 to 50 (red) were excluded from further data processing, B – changes in the class populations depending on the step of iteration. Dots are colored in accordance with a numbering color on the A panel

**Fig. 6:**
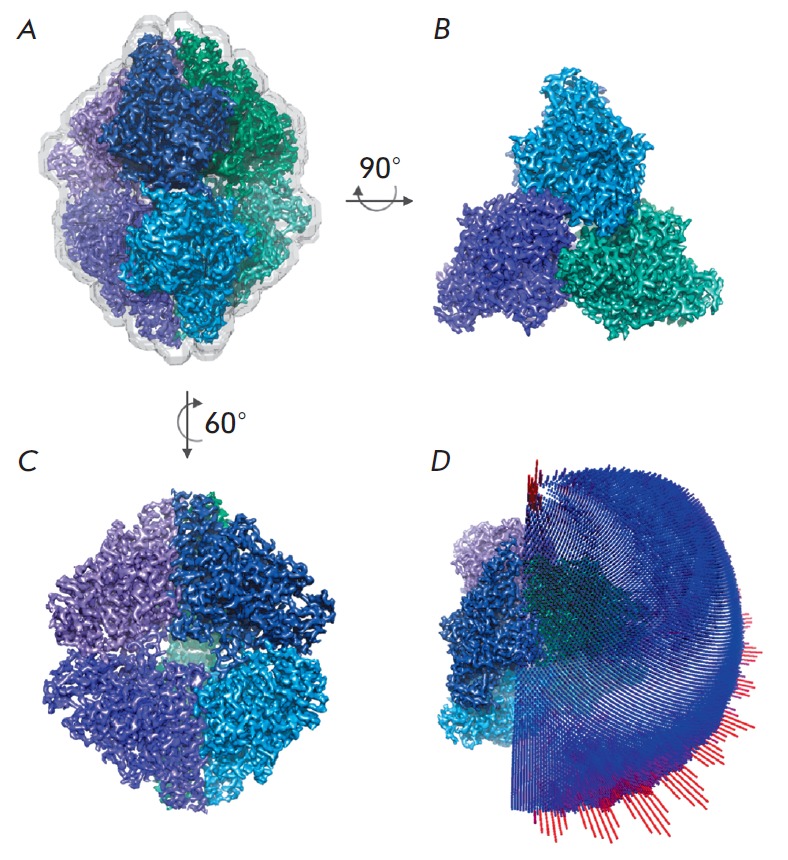
Final density map with the best resolution of 2.56 Å. A – the mask used for reconstruction and resolution assessment is shown by a gray semi-transparent isosurface; B–C – density maps in different projections, individual subunits of the protein hexamer are highlighted in colors; D – distribution of projections for the angle classification used

The symmetry-imposed refinement in the sixth step (*Fig. 2*) resulted in resolution improvement to 2.65 Å. In the seventh step, the same particle set (*Fig. 2*) was subjected to 3D classification [22, 23] into six classes (*Fig. 5*) without angular or translational searches, using the same mask according to a procedure described in [24]. In the eighth step (*Fig. 2*), the maps were repeatedly refined for each class. This approach allowed us to select a data subset composed of 33,891 particle images (median defocus was –0.86 μm, with the values varying from –1.48 to –0.18 μm, as estimated with Gctf), which corresponded to the third 3D class and yielded a map with the best resolution of 2.56 Å (*Fig. 5*). In order to increase the resolution, the steps 6–8 (*Fig. 2*) were carried out with imposition of D3 symmetry and using a binary mask (*Fig. 6A*) that was created by applying 5-pixel isotropic extension, smoothing the boundaries by 5 pixels, and using an isosurface threshold of 0.02.

**Structure refinement with REFMAC5**

The crystal structure of TvNiR at the best resolution of 1.4 Å (RCSB ID code is 3FO3) served as a starting model for the refinement. Solvent molecules, except for those corresponding to experimental density peaks, were removed from the dimer located in the asymmetric unit of the crystal. The TvNiR hexamer used for the refinement was generated by application of the appropriate symmetry operations. The refinement was performed with REFMAC5 [25] implemented in the CCP-EM suite [26].

A map of the third 3D class (*Fig. 5*), which had the best resolution, was used as the experimental cryo-EM map. To prevent the model from overfitting, the following approach was employed [27]. The images, from which the entire map was produced, were randomly divided into two subsets, and these subsets were used for the calculation of two independent ‘half maps’ and cross-validation. The high resolution of the experimental data allowed us to perform the refinement with no restraints, except for restraints on deviations of the bond lengths from the average value (jelly-body refinement [25]). To more correctly estimate steric constraints, hydrogen atoms were included in the refinement in fixed positions. After 30 refinement cycles, the fit of the refined model to the experimental density was visually inspected with Coot [28]. The influence of map sharpening/blurring on the refinement was assessed by performing a series of refinements with different degrees of blurring, from –150 to +150. The appropriate parameters for further refinement were selected based on the best Rf and FSC, which corresponded to a blur parameter of –80. In addition to the visual inspection, the quality of the refined model was validated with Molprobity [29]. The refined model was compared with the crystal structure of the protein using PDBeFOLD [30]. The Ramachandran plot analysis showed that the residues Gly285 and His361 of all subunits were in disallowed regions; however, these residues in all subunits had a well-defined electron density.

## DISCUSSION

The 3D classification revealed no significant structural heterogeneity in the sample at the resolution level achieved, but it made it possible to select a subset of particles containing information on high spatial frequencies and providing maximum resolution for the final cryo-EM map (*Fig. 6 B,C*).

**Fig. 7:**
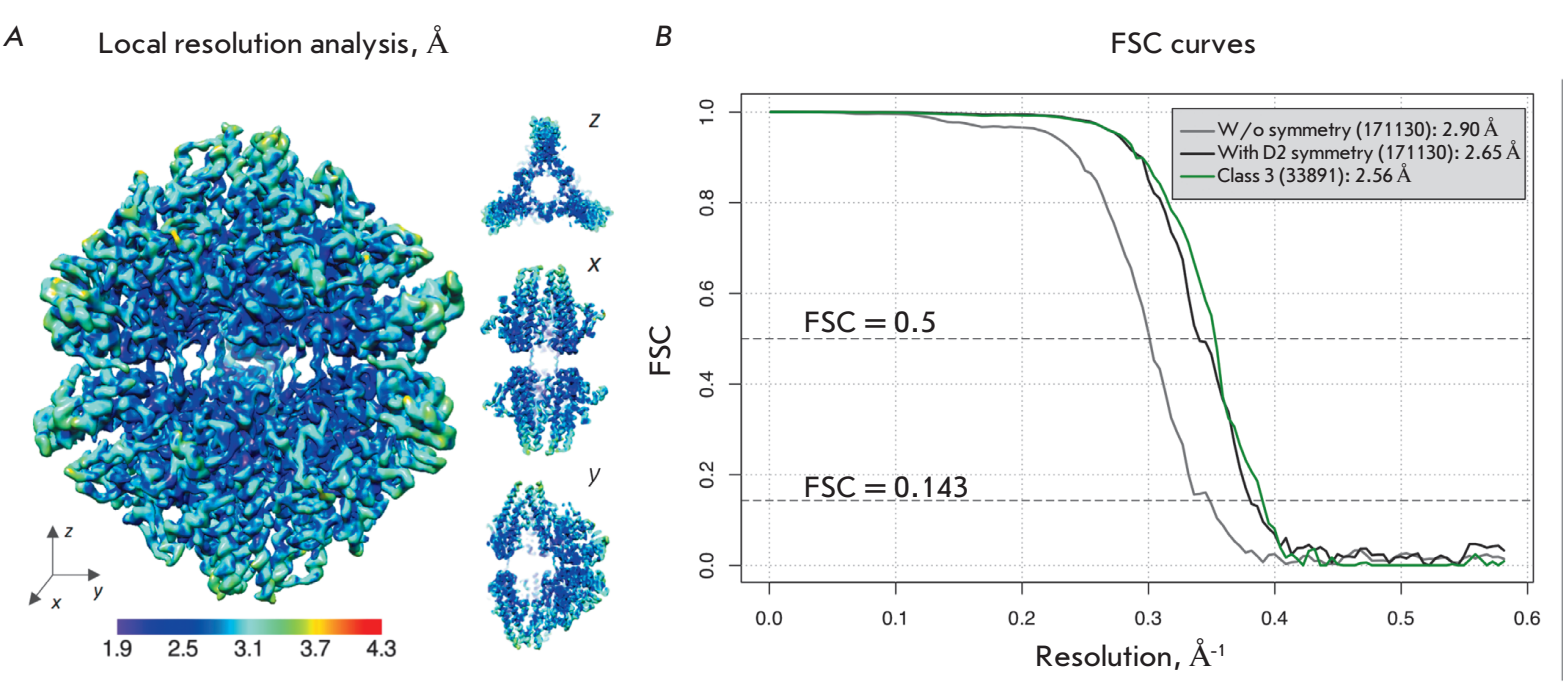
Resolution assessment. A – local resolution for the density map of the third 3D class analyzed with MonoRes [31]; B – Fourier shell correlation (FSC) curves for the density maps without imposition of symmetry (gray curve), with symmetry constraints for the complete set of particle images (black curve), and for a particle set belonging to the third class (green curve)

The distribution of projections for the angle classification, which was used in the refinement of the map without imposition of symmetry and the map for the third 3D class, is shown in *Fig. 6D*. It can be seen that the object has no preferred orientations in the amorphous ice layer. The assessment of the average resolution by the FSC = 0.143 and FSC = 0.5 criteria based on the results of postprocessing is shown in *Fig. 7*.

High symmetry of the object significantly simplifies the cryo-EM reconstruction, thereby compensating for a small number of projections with a rather high in formation limit for this experiment and a low signal-to-noise ratio due to the low molecular weight of the object. The results of the refinement in Relion 2.1 after 3D classification clearly show the influence of the data quality on the final resolution. When applying symmetry of the object, the structural model was constructed using a total of 34,000 particle images from the 3D class that had the highest resolution of 2.56 Å (*Fig. 5*).

**Fig. 8:**
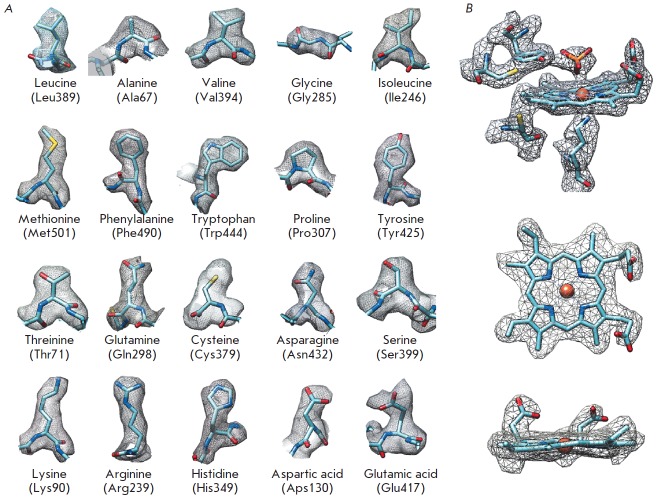
Quality of the experimental density for the third 3D class. A – for the side chains of selected residues of the refined model; B – for the active-site heme and its environment

The fitting of the enzyme X-ray structure (RCSB ID code 3FO3) to the experimental 2.56 Å resolution cryo-EM map and the subsequent refinement yielded a final structure with a Molprobity score of 2.55 and the following parameters: R_f_ = 28.70, FSC_average_ = 0.8679 (*Table*). The high-quality map allowed us not only to trace the polypeptide chain, but also to identify the side chains of residues (*Fig. 8A*), including the unique covalent bond between the active-site tyrosine and cysteine (*Fig. 8B*) and the side chains of residues Arg52, Arg316, and Lys456 on the surface of the enzyme molecule, which were invisible in the crystal structure. The density observed in the active site of the enzyme was identified as phosphate based on the composition of the buffer used for crystallization (*Fig. 8B*). The binding of inorganic anions is characteristic of the active site of TvNiR, which is well-known from X-ray crystallography [9, 10].

The superposition of the refined cryo-EM structure on the initial crystal structure shows that their backbone structures are, in general, similar. The side-chain orientations are also similar, except for some residues on the surface of the enzyme, e.g., Asp40, Arg51, Glu337, Glu341, *etc*., which may be attributed to their relatively high flexibility. The RMSD of the C_α_ atoms of these structures are not larger than 0.36 Å. Therefore, the cryo-EM structure of TvNiR is in good agreement with the crystal structure of the enzyme determined earlier by X-ray crystallography. The cryo-EM map was deposited in the Electron Microscopy Data Bank (EMDB) under the accession code EMD-0020.

## CONCLUSION

The structure of cytochrome *c *nitrite reductase from the bacterium *T. nitratireducens *was studied by cryo*- *EM single-particle analysis; a cryo-EM map with a 2.56 Å resolution was obtained, and the appropriate three-dimensional model was constructed. The optimal algorithm was found for data collection and processing in order to achieve high resolution. A comparison of the three-dimensional TvNiR structures determined by X-ray crystallography (1.40 Å) and cryo-EM (2.56 Å) revealed no significant differences. At the resolution level achieved by cryo-EM, TvNiR does not exhibit structural heterogeneity.

## Abbreviations

TvNiR: cytochrome c nitrite reductase from Thioalkalivibrio nitratireducens
cryo-EM: cryo-electron microscopy
CMOS: complementary metal-oxide semiconductor
CTF: contrast transfer function
FSC: Fourier shell correlation
